# Is subtotal gastrectomy feasible for the treatment of gastric stump cancer located at the anastomotic site after distal gastrectomy for benign lesions?

**DOI:** 10.1186/s12957-020-01821-y

**Published:** 2020-02-27

**Authors:** Fuhai Ma, Yang Li, Weikun Li, Wenzhe Kang, Hao Liu, Shuai Ma, Bingzhi Wang, Yibin Xie, Yuxin Zhong, Yingtai Chen, Liyan Xue, Yantao Tian

**Affiliations:** 1 Department of Pancreatic and Gastric Surgery, National Cancer Center/National Clinical Research Center for Cancer/Cancer Hospital, Chinese Academy of Medical Sciences and Peking Union Medical College, No. 17 Panjiayuan Nanli, Beijing, 100021 China; 2 Department of Pathology, National Cancer Center/National Clinical Research Center for Cancer/Cancer Hospital, Chinese Academy of Medical Sciences and Peking Union Medical College, Beijing, 100021 China

**Keywords:** Gastric stump cancer, Anastomotic site, Subtotal gastrectomy, Total gastrectomy

## Abstract

**Background:**

Total gastrectomy (TG) is a widely accepted procedure for treating gastric stump cancer (GSC). However, subtotal gastrectomy (SG) would benefit elective patients with GSC. The aim of this study was to clarify the safety and long-term prognosis of SG in treating GSC after distal gastrectomy for benign lesions.

**Methods:**

A total of 53 patients with GSC located at the anastomotic site or gastric body between May 1999 and December 2018 at our hospital were included. In total, 21 patients underwent SG, and the remaining 24 patients underwent TG. Clinicopathological data, operative data, and overall survival (OS) were compared.

**Results:**

The operative duration, estimated blood loss volume, and length of hospital stay were similar between the SG and TG groups. The postoperative complications were similar between the two groups, but no cases of anastomotic leakage were noted in the SG group. TG was associated with significantly more retrieved lymph nodes than SG (18.5 ± 11.5 vs. 10.7 ± 9.2; *p* = 0.017), while the number of metastatic lymph nodes did not differ between the groups (2.9 ± 3.5 vs. 1.9 ± 3.6; *p* = 0.329). The median survival time in the SG group was 81.0 months (95% confidence interval (CI), 68.906 to 93.094 months), which was similar to the 45.0 months (95% CI, 15.920 to 74.080 months) observed in the TG group (*p* = 0.236). Both univariate and multivariate analyses showed that tumor location and histological type were prognostic factors, while surgery type was not a prognostic factor. Further stratified analyses according to tumor location revealed that OS was not significantly different between the two groups among patients with tumors located at the anastomotic site, while OS in the TG group was significantly better than that in the SG group among patients with tumors located in the gastric body (*p* = 0.046).

**Conclusions:**

The results of the current study indicate that SG is a suitable alternative surgical procedure for GSC located at the anastomotic site after distal gastrectomy for benign lesions. The short-term outcomes and long-term prognoses of SG are comparable with those of TG.

## Background

Gastric cancer is the fifth most frequently diagnosed cancer and the third leading cause of cancer-related death globally [1, 2]. The incidence of gastric stump cancer (GSC) has been reported to represent 1–8% of all gastric cancer cases, and this number continues to increase [3–5]. GSC is characteristically considered a separate clinical entity, defined as adenocarcinoma arising in the gastric stump more than 5 years following an initial gastrectomy for benign disease [6]. Gastrectomy was frequently performed for benign ulcers two or three decades ago. However, the risk of GSC is closely linked to the interval after the initial gastrectomy [7]. Therefore, GSC will continue to be encountered by surgeons [4, 8].

In comparison with primary gastric cancer, GSC is commonly diagnosed at an advanced stage with a low rate of curative resection, resulting in a poor prognosis [9–11]. However, when GSC is resected curatively, there is no significant difference in the prognosis between GSC and primary gastric cancer [12–14]. Although there are no guidelines for the surgical treatment of GSC, total gastrectomy (TG) has been accepted as a standard procedure. In our hospital, we also perform subtotal gastrectomy (SG) of the gastric stump for patients with GSC located at the anastomotic site following distal gastrectomy for benign disease. To the best of our knowledge, only three studies have demonstrated that SG of the gastric stump is feasible for patients with early GSC [15–17]. All previous studies investigating the feasibility of SG for GSC were limited to the early stage of the disease.

In this retrospective study, we compared the outcomes of SG to those of TG for GSC following distal gastrectomy for benign disease to clarify the safety and long-term prognosis of SG.

## Methods

### Study patients

**Fig. 1:**
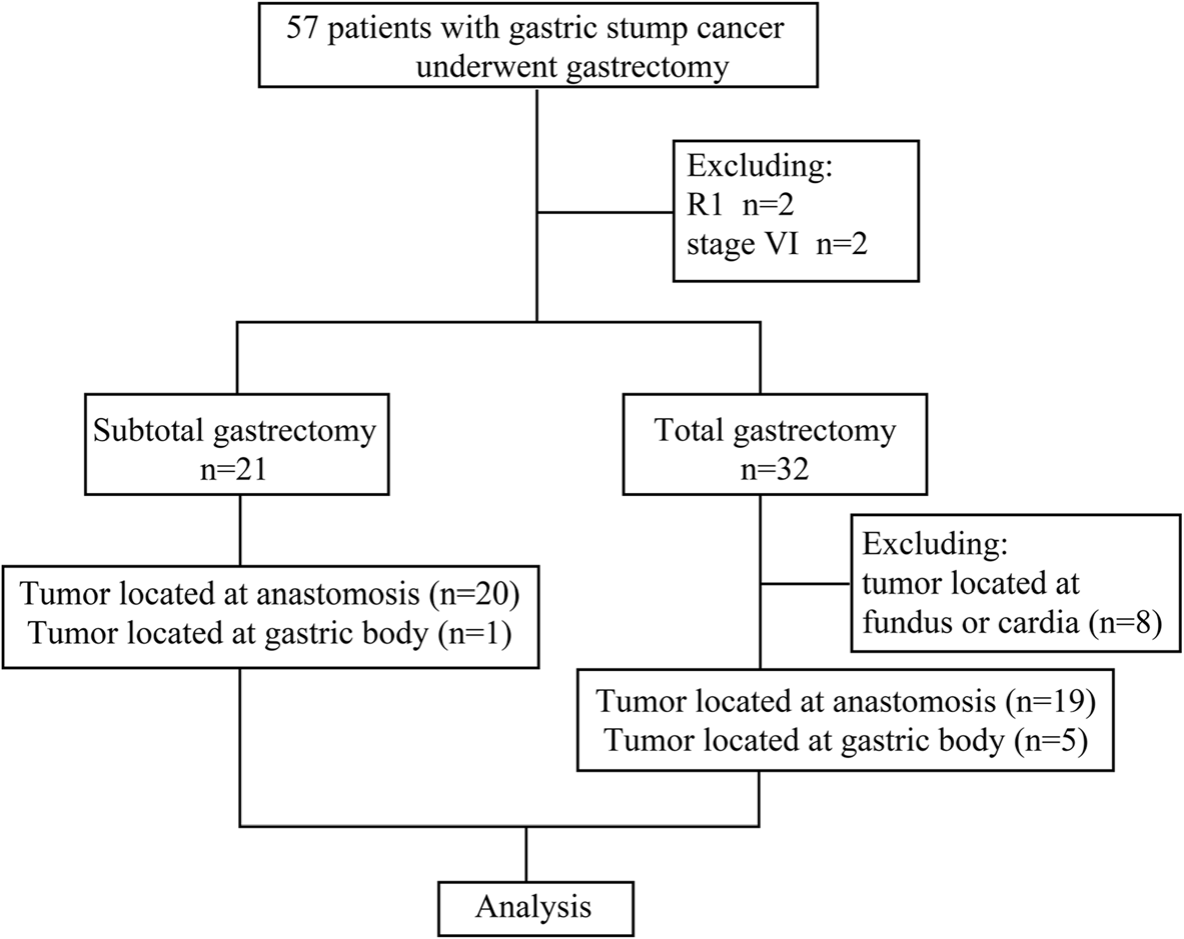
Study flow chart

A database search of patients who underwent surgery for GSC at the Cancer Hospital, Chinese Academy of Medical Sciences, between May 1999 and December 2018 was performed. GSC was defined as gastric cancer that occurred in the gastric stump at least 5 years after distal gastrectomy for benign lesions. Patients with recurrent malignant tumors after distal gastrectomy or metachronous gastric adenocarcinoma were excluded from the analysis. A total of 57 patients who underwent gastrectomy for GSC were identified, but four (two patients with R1 resection and two patients who underwent palliative resection) were excluded. Among the 53 patients, 32 and 21 patients underwent TG with radical lymph node dissection and SG with radical lymph node dissection, respectively. Because the indication of SG was the presence of a tumor in the gastric body and at the anastomotic site, eight patients with tumors located in the fundus and cardia of the stomach were excluded from the TG group. Finally, a total of 21 patients who underwent SG and 24 patients who underwent TG were analyzed (Fig. 1).

### Operative procedure

SG was defined as segmental resection of the distal gastric stump, including the site of anastomosis, along with lymph node dissection. Lymph nodes along the lesser curvature, the left gastric artery or the stump of the left gastric artery (if the left gastric artery was not preserved during initial distal gastrectomy), the splenic artery, the celiac axis, the superior margin of the pancreas, and the anastomotic duodenum or jejunum were usually dissected. Repeated Billroth II or Roux-en-Y procedures were usually used for reconstruction. TG for GSC was performed according to the conventional procedure with preservation of the spleen. In addition to the lymph nodes mentioned above for SG, group 2 and 4sa lymph nodes were also dissected in the TG procedure. The Roux-en-Y procedure was used for reconstruction after TG. The indications of SG for GSC were the presence of a tumor located at the anastomotic site or gastric body adjacent to the anastomosis and a sufficient proximal margin (> 5 cm from the anastomosis). Because of the effects of the primary disease and surgery, the anatomy and capacities of the gastric stump were different among different patients; thus, the ultimate choice of surgical procedure was made based on an individual case-by-case basis.

### Data collection and follow-up

Patient characteristics were obtained from a review of medical records. Demographic variables included age, sex, comorbidities, American Society of Anesthesiologists (ASA) physical status classification and body mass index (BMI). Clinicopathological characteristics included previous reconstruction, tumor location, tumor size, differentiation, gross type, and pathological stage. The short-term surgical outcomes, including the operative duration, estimated blood loss volume, number of intraoperative blood transfusions, postoperative complications, length of postoperative hospital stay, and number of dissected lymph nodes, were recorded. Pathological staging was determined based on the 8th edition of the Union for International Cancer Control (UICC) classification (pTNM). Histological type was classified as differentiated carcinoma (papillary adenocarcinoma, well-differentiated tubular adenocarcinoma, and moderately differentiated tubular adenocarcinoma) or undifferentiated carcinoma (poorly differentiated tubular adenocarcinoma, signet ring cell carcinoma, and mucinous adenocarcinoma).

Overall survival (OS) was determined as the period from the date of the operation until the date of death from any cause or until the end of the follow-up period. Follow-up was conducted mainly through telephone interviews. The last follow-up was conducted on June 30, 2019. This retrospective study was approved by the institutional review board of the Cancer Hospital of the Chinese Academy of Medical Sciences. The need for informed consent was waived due to the retrospective nature of the study, and the data were analyzed anonymously.

### Statistical analysis

The chi-square test and Fisher’s exact test were used for categorical variables, and Student’s *t* test was used for continuous variables. The cumulative survival rates were calculated using the Kaplan-Meier method, and the survival curves were compared using the log-rank test. The Cox proportional hazard model was used to verify independent prognostic factors. A *p* value < 0.05 was considered statistically significant. All statistical analyses were performed using SPSS version 22.0.

## Results

### Clinicopathological features

The clinicopathological characteristics of the included patients are shown in Table 1. There was no significant difference in age, sex, BMI, comorbidity rate, or ASA physical status classification between the two groups. The frequency of Billroth I and Billroth II reconstruction at the time of the initial surgery was approximately equal in the SG and TG groups. The mean time from the initial surgery to GSC diagnosis was comparable between the SG and TG groups (32.4 ± 7.4 vs. 31.7 ± 9.9 years; *p* = 0.788). With respect to tumor size, the tumors were larger in the TG group than in the SG group, but the difference was not significant. No significant difference was found between the two groups regarding the distribution of TNM stages. The ratio of patients who received postoperative chemotherapy was also similar between the two groups.

**Table 1:** Comparison of clinicopathological characteristics between the subtotal gastrectomy and total gastrectomy groups

Variable	Subtotal gastrectomy	Total gastrectomy	*P* value
	(*n* = 21) (%)	(*n* = 24) (%)	
Age (years)	62.9 ± 7.8	63.5 ± 8.5	0.720
Sex			0.670
Male	19 (90.5)	20 (83.3)	
Female	2 (9.5)	4 (16.7)	
BMI	21.2 ± 2.8	21.0 ± 3.4	0.885
ASA status			
I–II	14 (66.7)	16 (66.7)	1.000
III	7 (33.3)	8 (33.3)	
Comorbidity			
Any	5 (23.8)	2 (8.3)	0.255
Diabetes	1 (4.8)	0	
Cardiac	0	1 (4.2)	
Hypertension	4 (19.0)	1 (4.2)	
Previous reconstruction			1.000
Billroth I	1 (4.8)	1 (4.2)	
Billroth II	20 (95.2)	23 (95.8)	
Time interval (year, mean)	32.4 ± 7.4	31.7 ± 9.9	0.788
Histology type			0.376
Differentiated	12 (57.1)	10 (41.7)	
Undifferentiated	9 (42.9)	14 (58.3)	
Tumor location			0.193
Anastomotic site	20 (95.2)	19 (78.2)	
Gastric body	1(4.8)	5 (20.8)	
Tumor size (cm)	4.0 ± 1.4	5.2 ± 2.5	0.072
pT stage			0.830
T1a/1b	2 (9.5)	2 (8.3)	
T2	4 (19.0)	2 (8.3)	
T3	2 (9.5)	2 (8.3)	
T4a/4b	13 (61.9)	18 (75.0)	
pN stage			0.360
N0	13 (61.9)	9 (37.5)	
N1	4 (19.0)	5 (20.8)	
N2	2 (9.5)	5 (20.8)	
N3	2 (9.5)	5 (20.8)	
TNM stage			0.346
I	4 (19.0)	4 (16.7)	
II	8 (38.1)	5 (20.8)	
III	9 (42.9)	15 (62.5)	
Borrmann type			0.692
I	10 (41.7)	10 (41.7)	
II	5 (23.8)	3 (12.5)	
III	2 (9.5)	5 (20.8)	
IV	2 (9.5)	4 (16.7)	
Early stage	2 (9.5)	2 (8.3)	
Adjuvant chemotherapy			0.807
Yes	12 (57.1)	14 (58.3)	
No	7 (33.3)	9 (37.5)	
Missing	2 (9.5)	1 (4.2)	

*ASA* American Society of Anesthesiologists, *TG* total gastrectomy, *SG* subtotal gastrectomy (SG)

### Intraoperative and postoperative outcomes

Intraoperative and postoperative outcomes are shown in Table 2. The rate of combined resection was comparable between the SG and TG groups. There was no significant difference in the operative duration (189 vs. 190 min; *p* = 0.950), intraoperative estimated blood loss volume (256 vs. 350 ml; *p* = 0.182), number of blood transfusions (42.9% vs. 58.3%; *p* = 0.376) or length of postoperative hospital stay (13.4 vs. 15.3 days; *p* = 0.450). The number of harvested lymph nodes was greater in the TG group than in the SG group (18.5 ± 11.5 vs. 10.7 ± 9.2; *p* = 0.017), while the number of metastatic lymph nodes did not differ between the two groups (2.9 ± 3.5 vs. 1.9 ± 3.6; *p* = 0.329). The incidence of postoperative complications was 19.0% in the SG group and 20.8% in the TG group (*p* = 1.000); however, there were no cases of anastomotic leakage in the SG group. There were no cases of mortality in either group.

**Table 2:** Comparison of the surgical outcomes between the subtotal gastrectomy and total gastrectomy groups

Variable	Subtotal gastrectomy	Total gastrectomy	*P* value
	(*n* = 21) (%)	(*n* = 24) (%)	
Combined organ resection			0.250
Colorectal	3 (14.3)	3 (12.5)	
Spleen	0 (0)	4 (16.7)	
Other	1 (4.8)	1 (4.2)	
Operation time (min)	189 ± 55	190 ± 66	0.950
Intraoperative blood loss (ml)	256 ± 162	350 ± 314	0.182
Blood transfusion			0.376
Yes	9 (42.9)	14 (58.3)	
No	12 (57.1)	10 (41.7)	
Number of retrieved lymph nodes	10.7 ± 9.2	18.5 ± 11.5	**0.017**
Number of metastatic lymph nodes	1.9 ± 3.6	2.9 ± 3.5	0.329
Postoperative stay (days)	13.4 ± 5.8	15.3 ± 8.6	0.405
Total complications	4 (19.0)	5 (20.8)	1.000
Anastomotic leakage	0	3 (12.5)	
Intra-abdominal bleeding	1 (4.8)	1 (4.2)	
Intra-abdominal abscess	1 (4.8)	1 (4.2)	
Delayed gastric emptying	1 (4.8)	0	
Wound infection	1 (4.8)	0	
Mortality	0	0	

*TG* total gastrectomy, SG subtotal gastrectomy

### Survival results

**Fig. 2:**
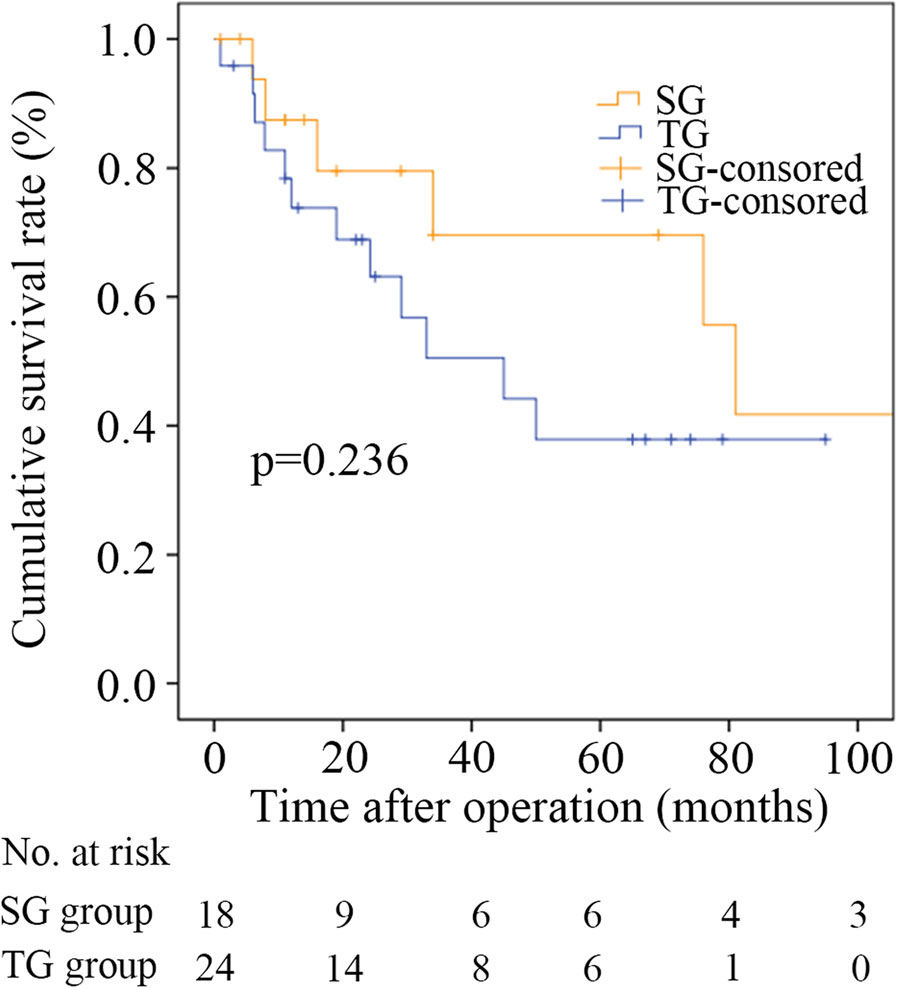
Overall survival curves of patients in the SG and TG groups. Overall survival was comparable in the SG and TG groups. Total gastrectomy (TG); subtotal gastrectomy (SG)

The median follow-up duration was 67.0 months (34.0 months for the SG group and 67.0 months for the TG group; *p* = 0.561). The median survival time in the SG group was 81.0 months (95% confidence interval [CI], 68.906 to 93.094 months), which was comparable with the 45.0 months (95% CI, 15.920 to 74.080 months) observed in the TG group (*p* = 0.236, Fig. 2).

**Fig. 3:**
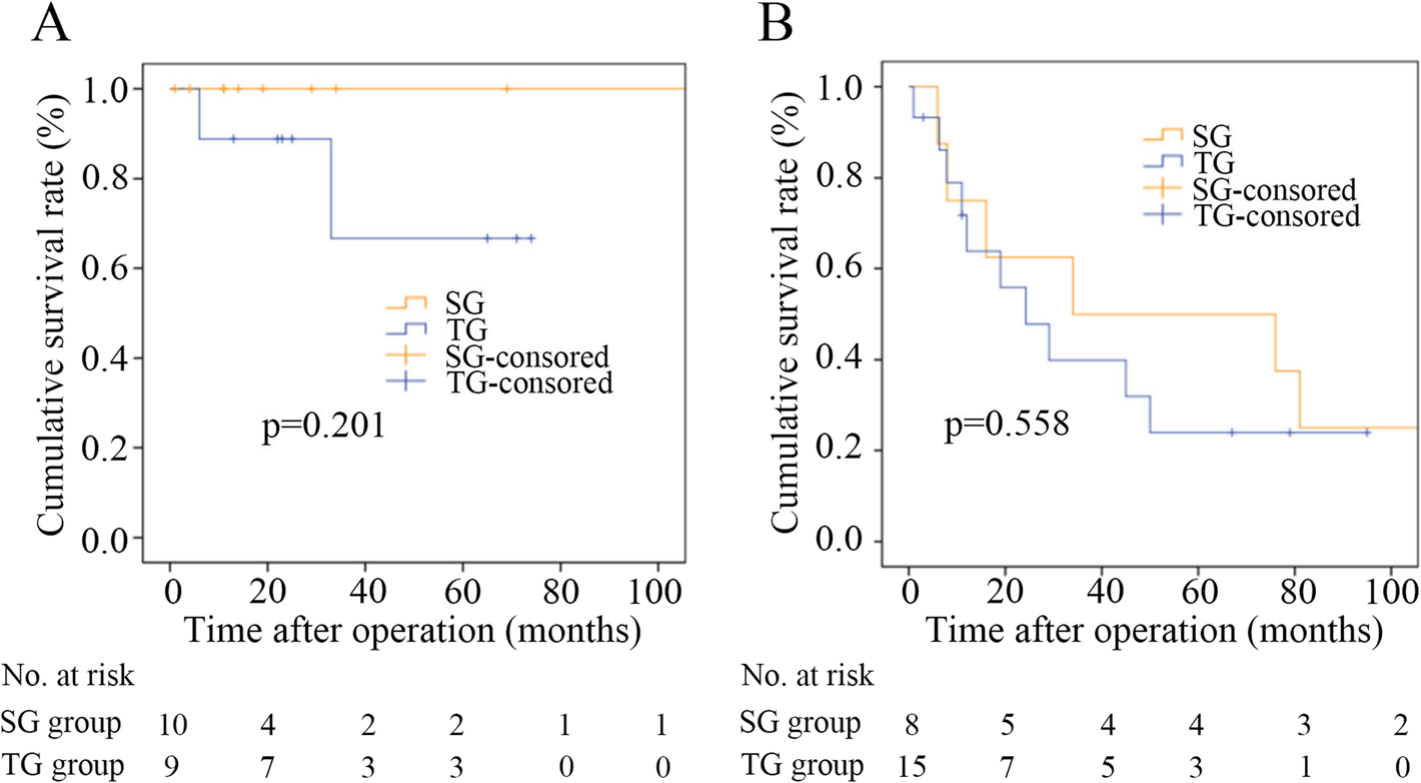
Stage-stratified survival curves of patients in the SG and TG groups. **a** For patients at stage I and II. **b** For subgroup analysis of stage III. Total gastrectomy (TG); subtotal gastrectomy (SG)

**Fig. 4:**
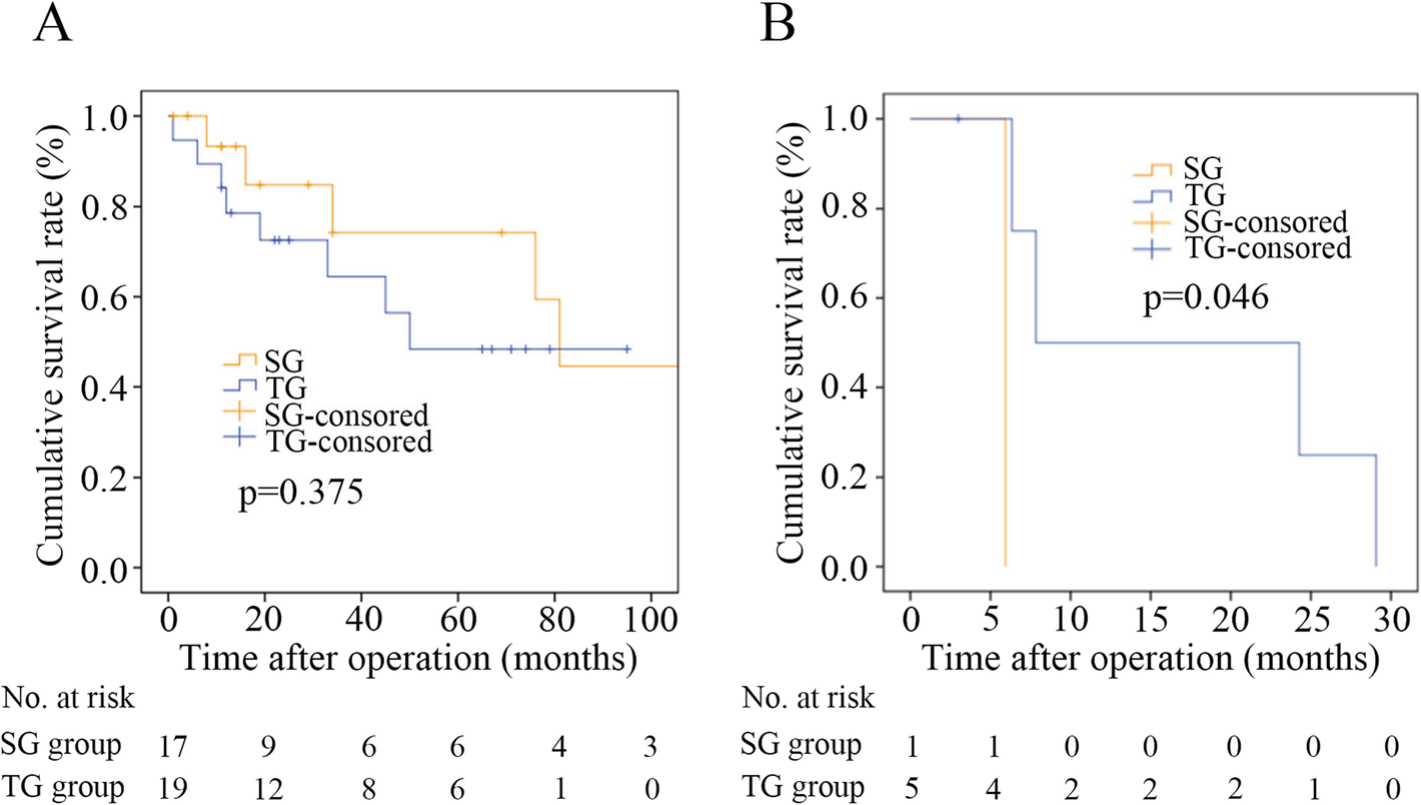
Stratified survival curves of patients in the SG and TG groups according to tumor location. **a** For patients with tumors located at the anastomotic site. **b** For patients with tumors located in the gastric body. Total gastrectomy (TG); subtotal gastrectomy (SG)

In the univariate and multivariate analyses, the two significant prognostic factors for OS were histology type and tumor site (Table 3). There were no significant differences in OS in the stage-stratified analyses: the median survival time was 34.0 months (95% CI, 0 to 117.156 months) in the SG group and 24.3 months (95% CI, 68.906 to 93.094 months) in the TG group among patients with stage III disease (*p* = 0.558), and the OS did not significantly differ between patients with stage I and stage II disease (*p* = 0.201) (Fig. 3). In the stratified analyses according to tumor location, OS did not significantly differ between patients with a tumor located at the anastomotic site (*p* = 0.375), while OS in the TG group was significantly better than that in the SG group among patients with a tumor located in the gastric body (*p* = 0.046) (Fig. 4).

**Table 3:** Univariate and multivariate analysis of overall survival

Variables	Univariate		Multivariate	
	Hazard ratio (95% CI)	*P* value	Hazard ratio (95% CI)	*P* value
Age: < 65 years vs. ≥ 65 years	1.096 (0.424–2.832)	0.850	–	–
Sex: male vs. female	1.006 (0.288–3.522)	0.992	–	–
Type of surgery: SG vs. TG	0.548 (0.200–1.502)	0.243	0.924 (0.308–2.788)	0.888
Combined resection: yes vs. no	1.303 (0.507–3.352)	0.583	-	–
Location: anastomotic site vs. gastric body	0.133 (0.042–0.425)	**0.001**	0.262 (0.074–0.933)	**0.039**
Histology type: undifferentiated vs. differentiated	3.597 (1.323–9.777)	**0.012**	2.820 (1.007–7.897)	**0.048**
Tumor size: > 5 cm vs. ≤ 5 cm	1.432 (0.552–3.719)	0.460	–	–
Interval: < 30 years vs ≥ 30 years	1.149 (0.444–2.977)	0.774	–	–
Stage: III vs. (II and I)	5.698 (1.301–24.961)	**0.021**	3.738 (0.808–17.295)	0.092
Chemotherapy: yes vs. no	1.320 (0.469–3.716)	0.599	-	–

*CI* confidence interval; *TG* total gastrectomy; *SG* subtotal gastrectomy

## Discussion

The ideal surgical method should not only achieve curative resection of the tumor with reduced morbidity and mortality but also result in a good long-term prognosis and favorable quality of life (QoL) for the patient [18]. In this study, we found that compared with TG for GSC located at the anastomotic site after distal gastrectomy for benign lesions, SG is associated with better short-term outcomes and equivalent long-term results, which in fact suggests that SG is a feasible and effective procedure for elective patients with GSC. To the best of our knowledge, this study is the first to compare SG and TG for GSC that is not limited to early-stage disease.

Previous studies have focused on the clinicopathological characteristics of GSC, and SG has been performed in only some cases [5, 7, 19]. In this study, SG was performed in 21 patients, and 20 of the 21 patients had tumors located at the anastomotic site of the gastric stump. The tumors in the SG group tended to be smaller than those in the TG group in our study. Therefore, a relatively small GSC lesion located at the site of anastomosis following distal gastrectomy for benign disease is often an indication for SG at our institution. Moreover, the current study demonstrated that compared with a tumor located in the gastric body, a tumor located at the anastomotic site was associated with better OS. OS did not significantly differ between patients in the SG group and those in the TG group in the subgroup analyses among patients with tumors located at the anastomotic site. Among patients with tumors located in the gastric body, the TG group had better OS than the SG group. Therefore, SG can be considered an alternative surgical procedure for GSC located at the anastomotic site.

Postoperative complications are an important factor regarding the safety and feasibility of a surgical procedure. Because of anatomical alterations and intra-abdominal adhesions, surgical treatments for GSC are difficult and are associated with relatively high rates of morbidity and mortality [7]. Yuichi Hosokawa et al. [15] and Tomoyuki Irino et al. [16] found a similar frequency of complications in the SG and TG groups for GSC. In the present study, we also found that the rate of complications was similar in the two groups; however, there were three cases of anastomotic leakage in the TG group and no cases of anastomotic leakage in the SG group. Previous studies have demonstrated that TG was independently associated with an increased risk of morbidity [20]. Moreover, Kim et al. [21] reported that the incidence of anastomotic leakage was significantly higher for TG than for SG. The reported rates of anastomotic leakage after TG vary from 4 to 15% [22]. In our study, the only three patients to exhibit anastomotic leakage were in the TG group, which may reflect an advantage of SG. Long-term prognosis is an important element for assessing oncological safety and a major concern in clinical practice. Specifically, SG can only be accepted as an alternative approach to TG if comparable long-term outcomes can be achieved. The results of our study suggest that SG for GSC located at the anastomotic site is associated with the same long-term outcomes as the traditional TG procedure, indicating that SG is feasible and safe from an oncological perspective.

Previous studies have reported that compared with TG, SG is associated with better short-term outcomes and similar long-term results in middle-third gastric cancer [23, 24]. Moreover, compared with SG patients, TG patients are expected to encounter more serious consequences, such as life-long vitamin B12 supplementation, more symptoms caused by food intolerance, and more alterations in dietary habits because of having a smaller food reservoir [25, 26]. Seung Lee et al. investigated long-term differences in QoL after SG and TG by comparing two groups and found an inferior QoL stemming from symptomatic and behavioral consequences of surgery in survivors 5 years after TG [27]. Regarding GSC, Yuichi Hosokawa et al. [15] compared SG and TG for GSC in 13 and 22 patients, respectively, and found that three patients in the TG group developed dumping syndrome, while no patients in the SG group developed dumping syndrome. Additionally, the hemoglobin and total protein levels were higher in the SG group than in the TG group 1 year after surgery. Although no studies have investigated QoL after SG for GSC, we believe that SG could serve as a function-preserving gastrectomy method that yields a better patient QoL.

During SG, lymph nodes, including lymph nodes in groups 1, 3, 4sb, 7, 8a, 9, 11p, and 12a and along the anastomotic duodenum or jejunum, were dissected, which is similar to the lymph node dissection performed in radical distal gastrectomy. For the TG procedure, in addition to the lymph nodes mentioned above, group 2 and 4sa lymph nodes were also dissected. Therefore, understandably, the total number of harvested lymph nodes was higher in the TG group than in the SG group. Many studies have demonstrated that an insufficient number of retrieved lymph nodes is independently associated with a poor prognosis, and patients with 15 or fewer retrieved lymph nodes exhibit a worse prognosis than those with 15 or more retrieved lymph nodes [28, 29]. However, the number of retrieved lymph nodes in GSC surgery is generally lower than the number retrieved in primary gastric cancer surgery because some perigastric lymph nodes are dissected during the initial operation. In the present study, the average number of retrieved lymph nodes was 10.7 and 18.5 in the SG and TG groups, respectively. In our study, OS did not differ between the two groups. Therefore, a low number of retrieved lymph nodes in SG does not necessarily imply insufficient treatment. The lymphatic pathway in GSCs differs from that in the original stomach. In addition to the left gastric artery and posterior gastric artery in the normal stomach, an important lymphatic flow pathway exists along the anastomotic site and jejunum [30]. During the TG and SG procedures, the lymph nodes in the anastomotic jejunal mesentery or the duodenum were also removed.

Nevertheless, this study has several limitations. First, the retrospective nature of this study indicates the potential for selection bias, and no information was available on the cause of death. Second, the number of patients enrolled was relatively small, mostly because of the rarity of the disease. Third, the overall median follow-up duration was relatively short. Despite these limitations, our study is the first to clarify the feasibility and efficacy of SG for GSC located at the anastomotic site after distal gastrectomy for benign lesions.

## Conclusions

In conclusion, the results of our study indicate that SG is a suitable alternative surgical procedure for GSC located at the anastomotic site after distal gastrectomy for benign lesions, with short-term outcomes and long-term prognoses comparable with those of TG. However, further studies with larger patient groups are necessary to reach a more definitive conclusion.

## Data Availability

The datasets supporting the conclusions of this article are available from the corresponding author upon request.

## Abbreviations

ASA: American Society of Anesthesiologists (ASA)
BMI: Body mass index (BMI)
OS: Overall survival (OS)
CI: Confidence interval (CI).GSC: Gastric stump cancer (GSC)
QoL: Quality of life (QoL)
SG: Subtotal gastrectomy (SG)
TG: Total gastrectomy (TG)
